# Poly(I:C) stimulation is superior than Imiquimod to induce the antitumoral functional profile of tumor‐conditioned macrophages

**DOI:** 10.1002/eji.201847888

**Published:** 2019-02-28

**Authors:** Akihiro Maeda, Elisabeth Digifico, Fernando T. Andon, Alberto Mantovani, Paola Allavena

**Affiliations:** ^1^ Humanitas Clinical and Research Center IRCCS Department of Innate Immunity and Inflammation via Manzoni 56 20089 Rozzano Milan Italy; ^2^ Humanitas University Via A. Manzoni 113 20089 Rozzano Milan Italy

**Keywords:** Imiquimod, Immunomodulation, Immunotherapy, Poly(I:C), Tumor‐associated macrophages (TAMs)

## Abstract

Macrophage plasticity is the ability of mononuclear phagocytes to change phenotype, function, and genetic reprogramming upon encounter of specific local stimuli. In the tumor microenvironment, Tumor‐Associated Macrophages (TAMs) acquire an immune‐suppressive and tumor‐promoting phenotype. With the aim to re‐educate TAMs to antitumor effectors, in this study, we used two immunestimulatory compounds: the TLR7 agonist Imiquimod (IMQ) and the TLR3 agonist Poly(I:C). To better mimic in vitro the response of TAMs, we used Tumor‐Conditioned Macrophages (TC‐Mϕ) differentiated in the presence of tumor cell supernatants. Our results show that TC‐Mϕ respond differently from conventional M2‐polarized macrophages. Upon stimulation with IMQ, TC‐Mϕ did not upregulate major histocompatibility complex (MHC II) molecules and unexpectedly expressed increased CD206. With both compounds, TC‐Mϕ produced higher levels of inflammatory cytokines than M2 macrophages. IMQ and Poly(I:C) differed in the types of regulated genes and secreted mediators. Reflecting their signaling pathways, only IMQ significantly induced IL‐1β and IL‐6, while only Poly(I:C) stimulated CXCL10, and both upregulated CCL5. Of note, using a novel cytotoxicity assay, Poly(I:C), but not IMQ, was effective in triggering the cytotoxic activity of TC‐Mϕ against cancer cells. Overall, the results demonstrate that Poly(I:C) stimulation of TC‐Mϕ is superior than IMQ in terms of macrophage re‐education toward antitumor effectors.

## Introduction

Among immune cells infiltrating the tumor microenvironment (TME), Tumor‐Associated Macrophages (TAMs) are the most abundant and their crosstalk with cancer cells and functional role have been the object of intense research over the past decades 1, 2. TAM precursors are circulating monocytes, which are continuously recruited at cancer tissues by tumor‐secreted factors, because their presence and activities in the TME are of advantage to tumor cells. In fact, it is now established that TAMs display a number of tumor‐promoting functions, which span from direct effects on tumor cells, such as stimulation of survival, proliferation and invasion, to indirect effects: switch‐on of the neo‐angiogenesis, protease activity, and continuous remodeling of the tumor stroma. Furthermore, TAMs have strong immunesuppressive functions and hamper the activity of T‐lymphocytes mediating antitumor immune responses 1, 3.

Several strategies have been implemented to target TAM in therapeutic settings. Clear evidence has been provided in experimental tumors that depletion of TAM results in inhibition of tumor growth and metastases 4. TAM targeting has been pursued with toxic substances like clodronate liposomes, antagonists to the CSF1 receptor or to chemokines and indeed improved the antitumor efficiency, especially when combined with chemotherapy, as observed in experimental settings 1, 5, 6, 7, 8. Our group reported that the registered antitumor compound trabectedin has a peculiar effect on immune cells and is selectively cytotoxic to monocytes/macrophages, inducing a caspase‐8‐dependent apoptosis 6. Proof‐of‐concept evidence in human studies has also been provided that inhibition or depletion of TAM is worth pursuing, especially in combinations with other therapeutic strategies 1.

An alternative approach to target TAM for therapeutic purposes is to exploit their functional plasticity and activate them, within the tumor tissues, to become cytotoxic effectors against cancer cells. Macrophages are highly plastic cells displaying different functional activities depending on the stimuli to which they are exposed. This characteristic has been described in the dichotomy of M1 and M2 macrophages, where M1s are classically activated effectors stimulated by bacteria and Th1 cytokines (e.g. LPS and IFN‐gamma), and M2 macrophages are alternatively activated by Th2 cytokines such as IL‐4. This dichotomy is likely to be too rigid for the complex biology of macrophages and the numerous stimuli to which they are exposed in vivo, nevertheless it served well to categorize their different phenotypes and functions exerted in diverse conditions. Along this dogma, M1 macrophages defend our body against bacterial infections and stimulate adaptive immune responses, while M2 macrophages are active against parasitic infections and have a major role in the healing of damaged tissues 9, 10.

M1‐macrophages are known to be cytotoxic against tumor cells 9. In the tumor context, TAMs have been profiled and almost all studies agree that they resemble more closely to M2‐polarized macrophages, rather than M1 11. We also compared TAM isolated from human ovarian cancer tissues with in vitro generated M1 and M2 macrophages, and found that TAM and M2 macrophages share a substantial proportion of their transcriptome 12.

Among strategies aimed to re‐educate TAM into antitumor effectors, a number of approaches have been pursued, for instance, the use of agonist antibodies triggering the immunostimulatory receptors CD40 and CD137 13, 14, 15, 16, 17, or compounds activating different TLRs 18, 19. TLR receptors recognize different pathogen‐associated (or damage‐associated) molecular patterns and activate appropriate immune responses to limit the danger. TLRs are expressed by several immune cells: DCs, NK cells, and macrophages, but also by some epithelial cells 20, 21. Among TLRs investigated for eliciting innate immune responses against cancer are TLR3, TLR7, TLR8, and TLR9, which are located intracellular in the endosome compartment and recognize pathogen‐derived nucleic acids. Different TLR agonists have been tested in experimental mouse tumors models 22, 23, 24, 25 and are being investigated in early clinical trials to assess their safety and efficacy in cancer patients, most frequently in combination with conventional or target therapies 26, 27, 28. Here, we have studied the TLR7 agonist Imiquimod (IMQ), one of the few approved by FDA for the treatment of nonmelanoma skin cancers 26, 29, and the TLR3 agonist Poly(I:C), a dsRNA analog under study in the clinic as adjuvant in antitumor vaccines 27, 30, 31, 32.

In most in vitro studies investigating immunemodulatory drugs, macrophage cell lines have been used such as THP1 and RAW cells, or M‐CSF‐1‐generated monocyte‐derived macrophages. These cells may differ from real TAM, which are conditioned by the complex tumor environment. We have previously demonstrated that human monocytes cocultured with tumor cells or with their conditioned media differentiate into Tumor‐Conditioned Macrophages (TC‐Mϕ), whose phenotype and gene profiling are similar to that of TAM isolated from human tumors 12. Here, we have used TC‐Mϕ to investigate the repolarizing ability of IMQ and Poly(I:C) and have evaluated changes in cell phenotype, gene expression, and cytokine production, as well as direct cytotoxic effect on cancer cells.

## Results

### Establishment of the immunomodulation protocol with IMQ and poly(I:C)

To investigate the immunomodulatory ability of IMQ and Poly(I:C) to repolarize human macrophages toward an M1‐like phenotype, we used IL‐4‐stimulated M2 macrophages, as well as TC‐Mϕ exposed to tumor cell supernatants for 6 days, as previously described 12. Along the study, we used TC‐Mϕ exposed to the conditioned medium of two pancreatic tumor cell lines (PANC1 and PT45), that were named accordingly: TC‐Mϕ (PANC1) and TC‐Mϕ (PT45).

In a first set of experiments, we defined the best treatment conditions; representative results are shown in Supporting Information Fig. 1B, 72‐h exposure to drugs was necessary to increase membrane major histocompatibility complex (MHC) II levels in flow cytometry, as shorter incubation time was not sufficient; effective drug concentrations were extrapolated from a wider dose‐response and set to 5–10 μg/mL for IMQ and 10–20 μg/ml for Poly(I:C), in the absence of macrophage toxicity; finally, M2 macrophages were used as reference population as M0 macrophages (stimulated only with rhM‐CSF) were much less responsive to drugs (Supporting Information Fig. 1B and not shown). Supporting Information Fig. 1C shows representative pictures of macrophage cultures stimulated with IMQ and Poly(I:C): both M2 macrophages and TC‐Mϕ acquired an activated morphology upon drug exposure.

**Figure 1:**
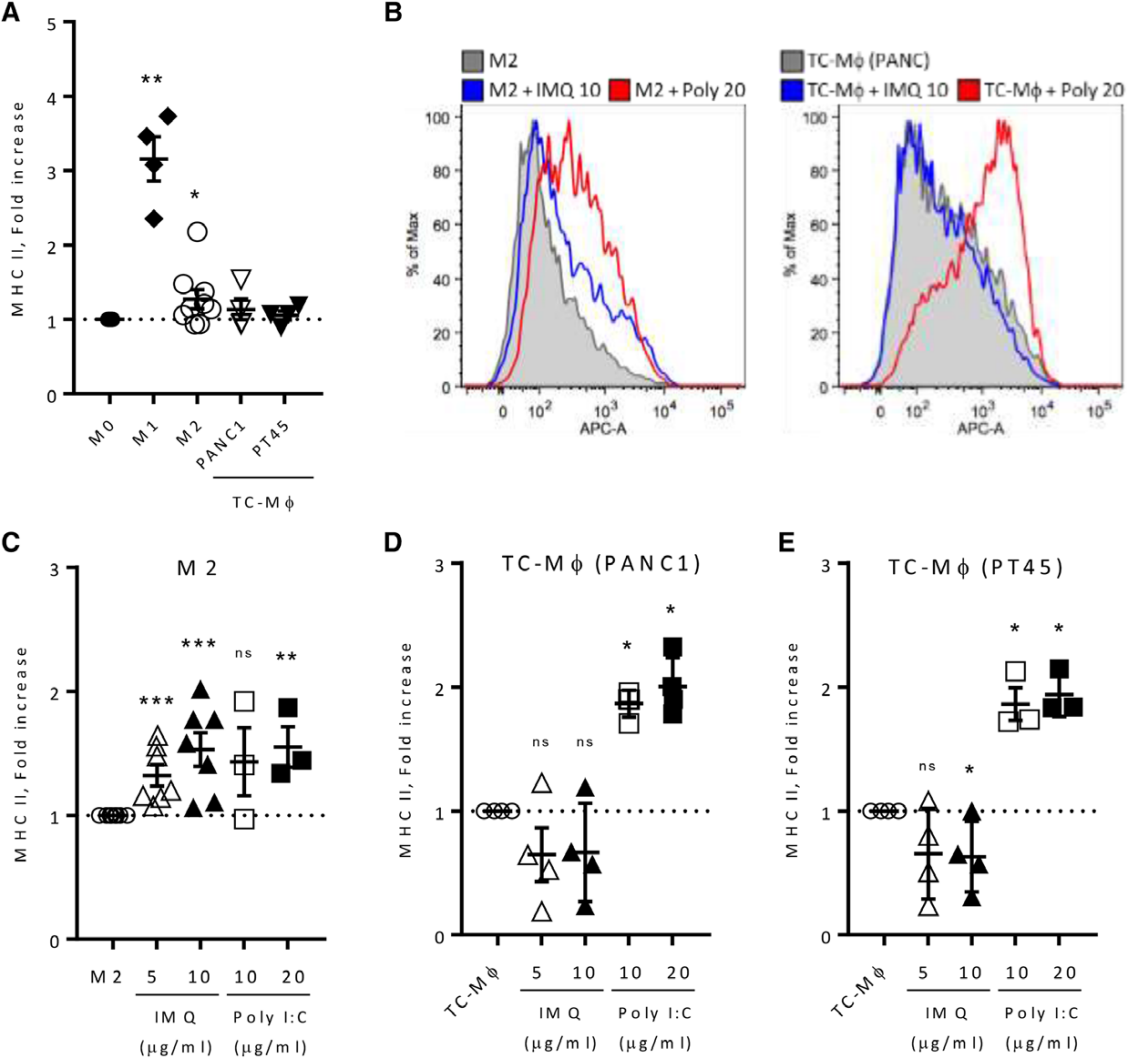
Expression of MHC II on M2‐macrophages and TC‐Mϕ treated with IMQ or Poly(I:C). Macrophages were in vitro differentiated by stimulating monocytes with 25 ng/mL of rhM‐CSF for 6 days, and then polarized with 100 ng/mL of LPS and 50 ng/mL of IFNγ (M1), 20 ng/mL of IL‐4 (M2) or medium (M0) for 24 h. TC‐Mϕ were prepared by stimulating monocytes for 6 days with 30% of tumor conditioned‐medium (from the tumor cell lines PANC1 or PT45). (A) Expression of MHC II analyzed by flow cytometry in differently polarized macrophages; the results are expressed as fold increase (Mean fluorescent intensity), relative to M0 macrophages; mean ± SEM are indicated from two to four independent experiments (total 3–7 donors); each symbol corresponds to a different blood donor). (B) Representative flow cytometry histograms of MHC II expression in M2 macrophages (left panel) and in TC‐Mϕ (right panel) treated with IMQ (10 μg/mL) or Poly(I:C) (20 μg/mL) for 72 h. (C–E) MHC II expression in M2 macrophages (C) and in TC‐Mϕ (D–E) treated with IMQ or Poly(I:C) for 72 h. Results are expressed as fold increase relative to unstimulated cells. *p < 0.03, **p < 0.002, and ***p < 0.0002 versus nontreated macrophages.

**Figure 2:**
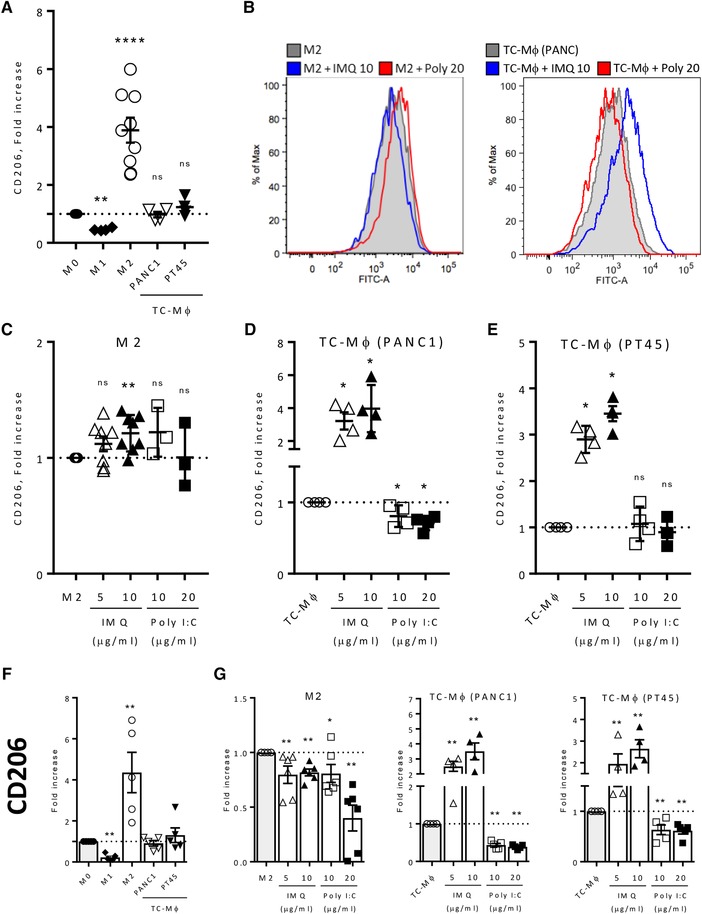
Expression of CD206 on M2‐macrophages and TC‐Mϕ treated with IMQ or Poly(I:C). Macrophages were prepared as detailed in the legend of Fig. 1. (A) Expression of CD206 by flow cytometry in differently polarized macrophages; the results are expressed as fold increase relative to M0 macrophages. (B) Representative flow cytometry histograms of CD206 expression in M2 macrophages (left panel) and in TC‐Mϕ (right panel) treated with IMQ (10 μg/mL) or Poly(I:C) (20 μg/mL) for 72 h. (C–E) CD206 expression in M2 macrophages (C) and in TC‐Mϕ (D‐E) treated with IMQ or Poly(I:C) for 72 h. Results are expressed as fold increase relative to unstimulated cells. (F) Gene expression analysis by qRT‐PCR of CD206 in differently polarized macrophages, and (G) in cells treated with IMQ or Poly(I:C) for 24 h. Mean ± SEM from two to three independent experiments (total 3–6 donors). *p < 0.03, **p < 0.002, and ****p < 0.0001 versus nontreated macrophages.

For the phenotype profile assessed by flow cytometry, Fig. 1A depicts an overview of different untreated macrophage populations: M0, M1 (LPS and IFNγ), M2 (IL‐4), and TC‐Mϕ, where, as expected, MHC II was elevated in M1 and not in M2 and TC‐Mϕ, while CD206 (Fig. 2A) was higher in M2 macrophages. The results are presented as fold relative to M0 macrophages. Expression of CD206 in TC‐Mϕ was substantially similar to that of M0 macrophages, but higher than in M1 macrophages.

### Modulation of cell membrane phenotype by IMQ and Poly(I:C)

Treatment with IMQ or Poly(I:C) clearly induced an upregulation of MHC II levels in M2‐polarized macrophages (Fig. 1B–C), but with both types of TC‐Mϕ, only Poly(I:C) was effective, while IMQ was not (Fig. 1B, D–E). Results are presented as fold relative to each untreated macrophage population. Both IMQ and Poly(I:C) were ineffective in downmodulating CD206 levels in M2 macrophages (Fig. 2B–C), while in TC‐Mϕ we found contrasting results: Poly(I:C) showed a tendency to reduce CD206, to a significant extent only in TC‐Mϕ (PANC1), while IMQ unexpectedly strongly upregulated CD206 on both types of TC‐Mϕ (Fig. 2B, D–E). In summary, Poly(I:C) was able to revert the phenotype of TC‐Mϕ toward an M1‐like phenotype, while the effect of IMQ was negligible on MHC II and contrary to expectations for CD206. Of note, RNA levels of CD206 exactly paralleled the phenotype results: strong increase with IMQ while significant decrease with Poly(I:C) (Fig. 2G).

### Modulation of gene expression by IMQ and Poly(I:C)

**Figure 3:**
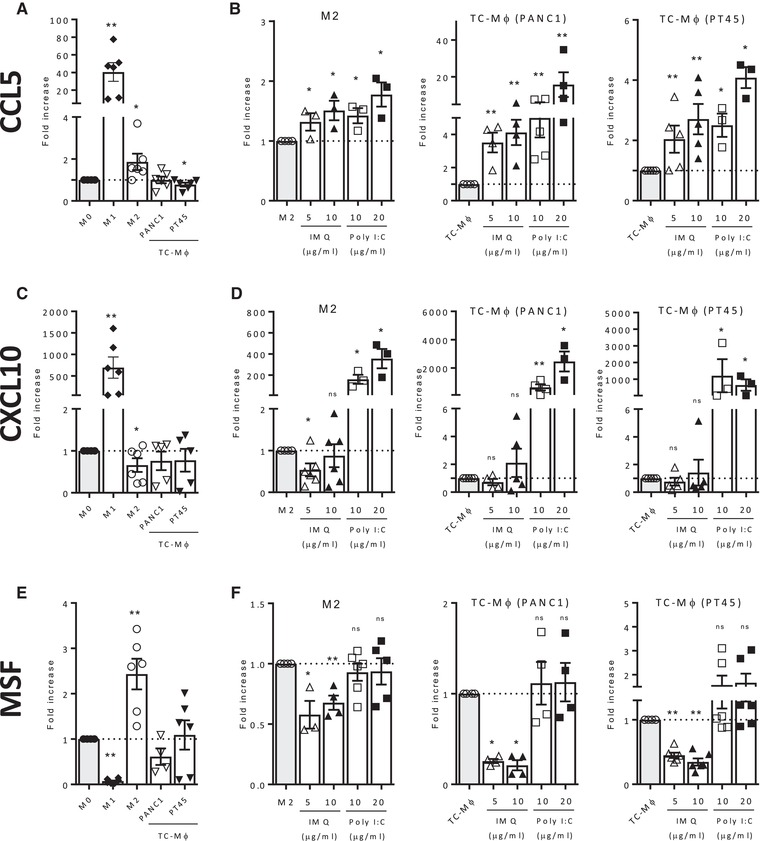
Gene expression analysis of M2‐macrophages and TC‐Mϕ treated with IMQ or Poly(I:C). Gene expression, analyzed by qRT‐PCR, in M0, M1, M2 macrophages, or TC‐Mϕ, which were prepared as detailed in the legend of Fig. 1 and  2. CCL5 (A), CXCL10 (C) and MSF (E). Results are expressed as fold over M0 macrophages. (B, D, and F) Gene expression in M2 macrophages or TC‐Mϕ treated with IMQ or Poly(I:C) for 24 h. Results are expressed as fold over untreated macrophages. Mean ± SEM from two to three independent experiments (total 3–6 donors). *p < 0.03 and **p < 0.002 versus nontreated macrophages.

We next determined the gene modulation upon drug treatment (24 h) on macrophages. Two typical M1‐related chemokines were investigated: CCL5 and CXCL10, and two M2‐related markers: CD206 (mentioned above), and a truncated version of fibronectin (migration stimulation factor, MSF) that we found highly expressed in M2 and TC‐Mϕ 12. Figure 3 panels A, C, and E show the results with the different populations of untreated macrophages. Both IMQ and Poly(I:C), stimulated the transcription of CCL5 in M2 macrophages and in TC‐Mϕ (Fig. 3B), while only Poly(I:C) was able to induce CXCL10 upregulation (Fig. 3D). Of interest, the increase of CXCL10 in TC‐Mϕ was much higher than in M2 macrophages (not so for the protein). The M2 prototypical marker MSF was significantly reduced by IMQ in M2 macrophages and in TC‐Mϕ, but not by Poly(I:C) (Fig. 3F).

**Figure 4:**
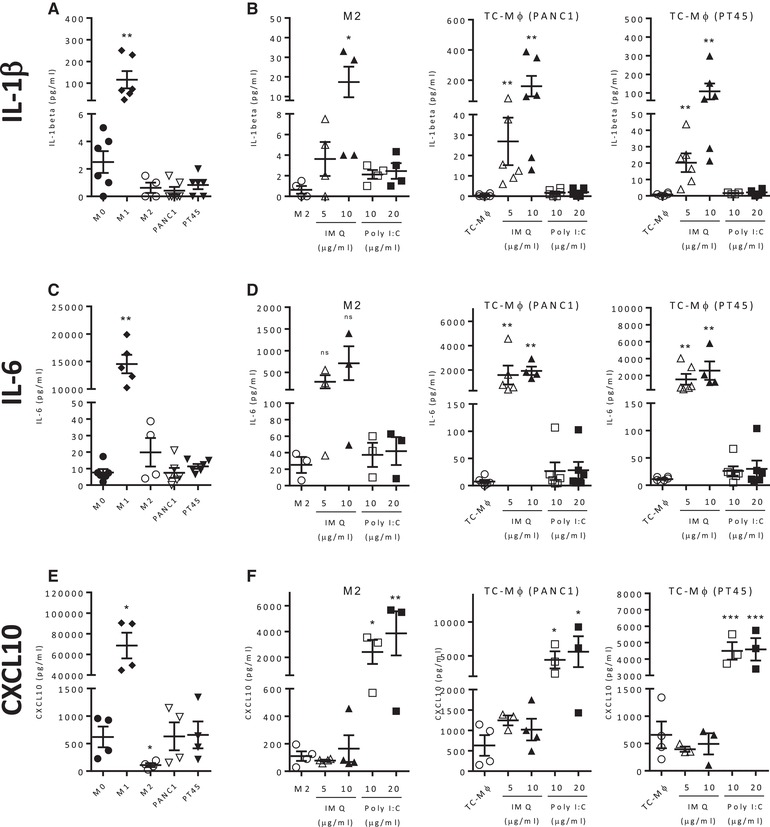
Cytokine/chemokine secretion by M2‐macrophages and TC‐Mϕ treated with IMQ or Poly(I:C). Cytokine/chemokine secretion measured by ELISA of IL‐1β (A), IL‐6 (C), and CXCL10 (E) from M0, M1, M2 macrophages, or TC‐Mϕ, which were prepared as detailed in the legend of Fig. 1 and  2. (B, D, and F) ELISA quantification from M2 macrophages or TC‐Mϕ treated with IMQ or Poly(I:C) for 24 h, IL‐1β (B), IL‐6 (D), or CXCL10 (F). Results are expressed as mean ± SEM from two to three independent experiments (total 3–6 donors). *p < 0.03, **p < 0.002, and ***p<0.0002 versus nontreated macrophages.

The release of soluble mediators was also tested by ELISA. As expected, IL‐1, IL‐6, and CXCL10 were produced by M1 macrophages, while M2 cells produced CCL17 (Fig. 4A, C, E, and Supporting Information Fig. 2A).

IMQ increased the production of IL‐1 and IL‐6 in both M2 macrophages and in TC‐Mϕ, while Poly(I:C) had no effect (Fig. 4B and D). On the other hand, in line with the mRNA results mentioned above, only Poly(I:C) stimulated the IFN‐responsive chemokine CXCL10 (Fig. 4F).

Finally, the chemokine CCL17 was not significantly modulated by the drugs (Supporting Information Fig. 2B).

In summary, gene modulation and cytokine/chemokine production showed a similar pattern in all types of macrophages, but the two drugs had some different effects: only IMQ stimulated IL‐1 and IL‐6 and repressed MSF, only Poly(I:C) upregulated CXCL10 and repressed CD206. A summary of these results is reported in Table 1.

**Table 1:** Summary of remodulation of M2/TC‐Mϕ by IMQ and Poly(I:C)

	MHC II	CD206	CCL5	CXCL10	MSF	IL‐1β	IL‐6
**on M2**							
IMQ	↑	↓	↑	–	↓	↑	↑
Poly(I:C)	↑	↓	↑	↑	–	–	–
**on TC‐Mϕ**							
IMQ	–	↑↑	↑	–	↓	↑	↑
Poly(I:C)	↑↑	↓	↑	↑	–	–	–

The gene expression and protein expression on stimulated M2/TC‐Mϕ were indicated as increase (↓), decrease (↑), or no change (–).

### In vitro cytotoxic activity of repolarized macrophages toward tumor cells

The crucial antitumor function of M1‐re‐educated macrophages is to become cytotoxic against tumor cells. Therefore, we set up a simple in vitro cytotoxicity assay where tumor cells stained with CellTrace were cocultured with macrophages (ratio: 10:1) for 48 h. At the end, all cells were trypsinized and analyzed in flow cytometry to enumerate live tumor cells.

**Figure 5:**
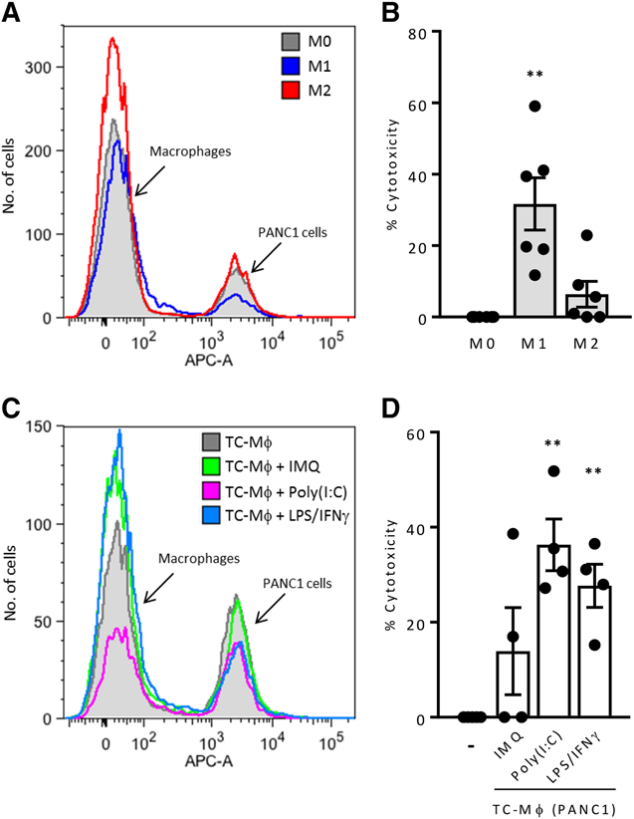
Cytotoxic activity on tumor cells of TC‐Mϕ treated with IMQ or Poly(I:C). The indicated macrophage populations were untreated or stimulated with IMQ (10 μg/mL) or Poly(I:C) (20 μg/mL) for 24 h and then coincubated with fluorescent labeled PANC1 tumor cells for 48 h. Fluorescent surviving tumor cells were quantified by flow cytometry. (A,C) Representative flow cytometry histograms. (B,D) Global analysis with the results from three independent experiments (total 3–6 donors). The results are expressed as % Cytotoxicity relative to M0 macrophages (B) or untreated TC‐Mϕ (D). **p < 0.002 versus control.

Figure 5A and B shows that classical M1 macrophages significantly exhibited cytotoxicity against tumor cells while M2 macrophages and untreated TC‐Mϕ (Fig. 5C and D) did not. Treatment with Poly(I:C) stimulated significant cytotoxicity of TC‐Mϕ up to 40%, while IMQ was modestly effective (Fig. 5C and D). Furthermore, TC‐Mϕ stimulated with LPS + IFN‐γ also showed similar levels of tumor killing.

## Discussion

In this study, we have used two TLR agonists known to have immune‐stimulatory properties: IMQ, a TLR7 ligand already approved for clinical use in dermatological cancers, and Poly(I:C), a TLR3 ligand, currently under investigation in early clinical trials as anti‐tumor vaccine adjuvant 28, 30, 33, 34, 35, 36. As responder cells, we employed primary human macrophages conditioned by tumor cells during their differentiation period (TC‐Mϕ), such macrophages are more representative of TAMs in the TME and share a similar global RNA profiling with human TAMs, as previously reported 12. The direct comparison (same donors) of TC‐Mϕ and IL‐4‐stimulated macrophages used as M2‐prototypic cells, indeed, showed some interesting differences in response to the drugs. For instance, IMQ was able to significantly upregulate MHC II molecules in M2 macrophages but was totally ineffective in TC‐Mϕ. On the other hand, IMQ strongly induced the expression of CD206 in TC‐Mϕ and not, or to a little extent in M2 cells. This unexpected upregulation of the Mannose Receptor is in line with the findings in a murine model of psoriasis‐like inflammatory disease, where skin macrophages in IMQ‐treated mice had higher expression of CD206 37. Mannose receptor upregulation is probably due to the IMQ‐induced production of IL‐10 38, which is known to be a major stimulus for this receptor 39.

TC‐Mϕ also differed from M2 cells because they were more efficient in producing higher amounts of inflammatory mediators such as IL‐1, IL‐6, CCL5 and CXCL10. This finding, observed with both types of tumor‐conditioned media (from PANC1 and PT45 tumor cells), indicate that macrophages exposed to tumor cell supernatants appear to be more primed/stimulated than macrophages receiving recombinant M‐CSF and IL‐4. This result is interesting because it demonstrates that macrophages directly influenced by tumor‐derived factors, are not anergic cells and are amenable to be re‐educated.

Overall Poly(I:C) performed better than IMQ as macrophage modulator. Poly(I:C) treatment caused a clear‐cut change in cell phenotype by increasing MHC II molecules in both M2 and TC‐Mϕ; CD206, instead, had only a slight decrease in the expression and only with TC‐Mϕ (PANC1), but mRNA levels were significantly reduced in both TC‐Mϕ populations. This difference in protein and mRNA modulation is likely due to a relatively long half‐life of the receptor on the cell membrane.

Modulation of cytokine production was clearly distinct with the two drugs. IMQ stimulated the proinflammatory mediators IL‐1, IL‐6, and CCL5, while Poly(I:C) did not trigger IL‐1, IL‐6, but rather stimulated the production of the T cell‐attracting chemokine CXCL10, and CCL5, as well. It is known that the two compounds show distinct signaling pathways: IMQ‐TLR7 triggers NF‐κB via the adaptor protein MyD88 resulting in the activation of an inflammatory cascade, while Poly(I:C)‐TLR3 signals through the TIR‐domain‐containing adaptor protein (TRIF) leading to IFN type I production and its target genes. Notably, we confirmed that IMQ, but not Poly(I:C), induced phosphorylated Iκ‐Bα (P‐Iκ‐Bα) (Supporting Information Fig. 3). Furthermore, P‐Iκ‐Bα was higher in TC‐Mϕ, than in M2 macrophages also after LPS stimulation (Supporting Information Fig. 3); this finding is in line with the higher levels of cytokines/chemokines produced by TC‐Mϕ (Fig. 4). Nevertheless, it is surprising that the cytokine pattern was so clear‐cut distinct, because it was reported that both drugs eventually fully activate NF‐kB 22, 40. As most studies looked at APCs, especially DCs, and as gene modulation appears to be cell‐dependent, it is possible that purified macrophages behave differently. The cytokine network is also important; in fact Muller et al reported that the response to various TLR ligands differed if specific additional cytokines, primarily, IFNγ was available to macrophages 41.

Previous studies reported that the potential antitumoral effects of TLR agonists were mediated through activation of innate immunity, as well as by increased recruitment of T‐cells within tumors. In the perspective of re‐educating TAM in an antitumor mode, the ability to recall T‐cells in the TME is certainly more beneficial than producing inflammatory mediators, which may positively impact on cancer cell proliferation and treatment resistance 42. Though the ability of IMQ to produce CCL5, should also be considered as an important determinant of T‐cell recruitment 43.

Another difference noted between Poly(I:C) and IMQ is on the regulation of MSF, which was reduced only by IMQ. This truncated isoform of fibronectin is preferentially produced by M2‐polarized and TC‐macrophages, while gene transcription is strongly inhibited in M1 cells, as confirmed here and previously demonstrated by our group 12. MSF is a good marker for distinguishing differently polarized macrophage populations. This very reactive isoform of fibronectin has potent chemoattractant ability for myeloid and tumor cells 12, 44 and also promotes angiogenesis 45. Therefore, IMQ is indeed able to counteract the M2‐like polarization of macrophages by inhibiting MSF expression, especially in TC‐Mϕ.

The ultimate goal of TAM re‐education is to stimulate their cytotoxic activity against cancer cells, with the hope to directly eliminate them. We have set up a simple nonradioactive cytotoxicity assay in vitro to detect macrophage‐mediated killing of tumor cells. While IMQ‐stimulated TC‐Mϕ showed only modest killing, Poly(I:C) induced more robust cytotoxic macrophages.

Overall our results offer insights into the response of TC‐Mϕ to TLR agonists and point to select Poly(I:C) as more potent immunemodulator to redirect the functional profile of TC‐Mϕ.

## Materials and methods

### Cells and tumor conditioned medium

Human pancreatic carcinoma cell lines PANC1 and PT45 were cultured in RPMI 1640 (Lonza, Basel, Switzerland) supplemented with 10% Fetal Bovine Serum (FBS). Once grown to 90% of confluence, media were discarded, and flasks were rinsed two times with saline solution. Cells were then incubated with fresh RPMI supplemented with 5% FBS for 24 h; the tumor conditioned (TC) medium was collected and the supernatant was stored at −20°C. All cell lines were routinely checked for Mycoplasma contamination.

### Human primary macrophages and TC‐Mϕ differentiation

Human primary monocytes from blood of healthy donors were purified through density gradients, as described in previous report 20. Briefly, human monocytes were obtained from normal blood donor buffy coats by a two‐step gradient centrifugation, first by Lympholyte‐H Cell Separation Media (Sigma–Aldrich, Milan, Italy) and then by Percoll (GE Healthcare, Milan, Italy). M0‐macrophage and TC‐Mϕ were obtained by culturing 10^6^ cell/mL monocytes for 5 days in 5% FBS/RPMI 1640 supplemented with 25 ng/mL of recombinant human M‐CSF (rhM‐CSF; PeproTech, Milan, Italy) or in the presence of 30% of TC medium. M1 macrophages were polarized by stimulating M0 macrophages with LPS (100 ng/mL) (PeproTech) and IFN‐g (50 ng/mL) (PeproTech) for 24 h, and M2 macrophages were polarized by IL‐4 (20 ng/mL) (PeproTech) for 24 h.

### Drug preparation and treatment

IMQ (Sigma) was dissolved in DMSO and warmed at 60°C for 15 min, and Poly(I:C) (Invitrogen, Milan, Italy) was prepared dissolving in water at 65°C for 10 min. For drug treatment, the differentiated macrophages were treated with the indicated concentrations of drugs in RPMI medium for indicated time.

### Flow cytometry

In vitro‐differentiated macrophages were analyzed by flow cytometry on FACSCanto II instrument (BD Biosciences, Milan, Italy). For staining, cells were washed and resuspended in FACS buffer (PBS 1% BSA). APC‐mouse anti‐human HLA‐DR (MHC class II), FITC‐mouse antihuman mannose receptor CD206, were obtained from BD Biosciences. The data were analyzed by FACS Diva software (BD Biosciences). For all samples, the analysis has been done with the first gating with FSC_hight_ and SSC_area_ for the macrophages, then gated with SSC_width_ and SSC_area_ to remove doublet cells (Supporting Information Fig. 1A). The gated cells were plotted on APC (MHC II) or FITC (CD206) and analyzed for mean fluorescent intensity (Mean FI).

### Measurement of secretory cytokines

Cytokine production was measured by commercially available ELISA kits (IL‐1β, IL‐6, CXCL10, CCL17) according to the manufacturer's instructions (R&D Systems, Space Import, Milan, Italy), using the supernatants collected after 24‐h treatment.

### Quantitative real‐time PCR (qRT‐PCR)

Total RNA extraction from the treated macrophages was performed with Trizol (Invitrogen, Milan, Italy). cDNA was synthesized by random priming from 1 mg total RNA with the High‐Capacity cDNA Reverse Transcription kit (Applied Biosystems, Monza, Italy) according to the manufacturer's instructions. Real‐Time PCR was performed using SYBR Green dye and QuantStudio 7 Flex Real Time PCR Systems (Applied Biosystems). The sequences of primer pairs specific for each gene (Sigma) were designed with Primer Express Software (Applied Biosystems) and were as follows: hGAPDH; 5′‐AGA TCA TCA GCA ATG CCT CCT G‐3′ and 5′‐ATG GCA TGG ACT GTG GTC ATG‐3′, hCCL5; 5′‐TGC ATC TGC CTC CCC ATA TT‐3′ and 5′‐ GAC CTT GCC ACT GGT GTA GAA A‐3′, hCXCL10; 5′‐GGA AGC ACT GCA TCG ATT TTG‐3′ and 5′‐CAG AAT CGA AGG CCA TCA AGA‐3′, hCD206; 5′‐ GGA GTG ATG GTT CTC CTG TTT‐3′ and 5′‐ CCT TTC AGC TCA CCA CAG TAT T‐3′, hMSF; 5′‐GCA TTG CCA ACC TTT ACA GAC‐3′ and 5′‐TTT CTG GGT GGG ATA CTC AC‐3′. A total of 2 μL cDNA was used as the template; 12.5 μL SYBR Green PCR Master Mix (Applied Biosystems) was mixed with template and primers. The total reaction volume was 25 μL. Cycling conditions were 10 min at 95°C, 40 cycles of 15 s at 95°C, and 1 min at 60°C. Experiments were performed in triplicate for each sample. mRNA was normalized to GAPDH mRNA by subtracting the cycle threshold (CT) value of GAPDH mRNA from the Ct value of the gene (∆CT). Fold difference was calculated by comparing the ∆CT with the ∆CT of nontreated M2 or TC‐Mϕ.

### Cytotoxicity of repolarized macrophages on tumor cell line

The primary monocytes isolated from human healthy donor was stimulated with M‐CSF in 5% FBS supplemented RMPI medium for 5 days, then polarized with TC from PANC1 for TC‐Mϕ for 24 h. The polarized macrophages were stimulated with IMQ or Poly(I:C) with indicated concentration or LPS/IFN‐γ for 24 h. After repolarization, the cells were coincubated with 25,000 cells of PANC1 cells, which is stained with CellTrace Far Red (Invitrogen), for 2 days. The cells were trypsinized and fixed for flow cytometry analysis using FACScanto II instrument. For the flow cytometry analysis, the number of events, which has high intensity of fluorescent (PANC1), were counted, but not low fluorescent intensity (macrophages) for 45 s acquisition time. The values were normalized by the nontreated TC‐Mϕ and calculated.

### Statistical analysis

Data are expressed as mean ± standard error (SEM) of 3–7 independent experiments (as indicated). Statistical analysis was performed using Graph Pad Prism 7. Significant difference between nontreated and treated samples was analyzed by two‐tailed *t*‐tests (Mann‐Whiteny test).

### Western blotting

The differentiated macrophages were treated with IMQ (10 μg/mL), Poly(I:C) (20 μg/mL), or LPS (1 μg/mL) for 30 min, then cells were lysed in Radioimmunoprecipitation (RIPA) buffer (50 mM Tris‐HCl pH 7.5, 150 mM NaCl, 5 mM EDTA, 1% NP‐40, 0.5% SDS, 1 mM Na3VO4, and Complete Protease Inhibitor Cocktail (Roche Diagnostic, Indianapolis, IN). Total proteins were measured by DC TM Protein Assay, according to manufacturer's instructions (Bio‐Rad Laboratories, Inc., Berkeley, CA).

Western blot analysis for Phospho‐Iκ‐Bα (mouse monoclonal anti‐Phospho‐Iκ‐Bα (Ser32/36), 1:1000; Cell Signaling Technology) was performed after loading 5 μg of cell lysate/lane on NuPAGE 4–12% Bis‐Tris Protein Gels (Thermo Fisher Scientific, Waltham, MA). After the anaylsis of Phospho‐Iκ‐Bα, the membrane was stripped and blotted with mouse monoclonal anti‐Iκ‐Bα (L35A5) (1:1000; Cell Signaling Technology). Optical density values were internally normalized using mouse monoclonal anti‐Vinculin (1:2000; Sigma‐Aldrich) and further corrected for the value of controls considered equal to 1.

## Conflicts of Interest

The authors declare no financial or commercial conflicts of interest.

**Abbreviations**
CCL: chemokine (C‐C motif) ligand
CXCL: C‐X‐C motif chemokine ligand
IFNγ: interferon gamma
IMQ: Imiquimod
MHC II: major histocompatibility complex class II
MSF: migration stimulation factor
TAMs: tumor‐associated macrophages
TC‐Mϕ: tumor‐conditioned macrophages
TME: tumor microenvironment

## Supporting information


**Figure S1**. Dose‐response and kinetics of IMQ on differently polarized human macrophages
**Figure S2**. CCL17 secretion by M2‐macrophages and TC‐Mϕ treated with IMQ or Poly(I:C).
**Figure S3**. Phosphorylation of Iκ‐Bα upon treatment with IMQ and Poly(I:C).
