# Supplementary material for: Poly(I:C) stimulation is superior than Imiquimod to induce the antitumoral functional profile of tumor‐conditioned macrophages
Source: Eur J Immunol. 2019 Feb 28;49(5):801–11. doi: 10.1002/eji.201847888 (PMC6563448; doi:10.1002/eji.201847888)
Supplement: Supplementary file 1 — Figure S1. Dose‐response and kinetics of IMQ on differently polarized human macrophages Figure S2. CCL17 secretion by M2‐macrophages and TC‐Mϕ treated with IMQ or Poly(I:C). Figure S3. Phosphorylation of Iκ‐Bα upon treatment with IMQ and Poly(I:C). [file EJI-49-801-s001.pdf]

European Journal of Immunology

Supporting Information
for
DOI 10.1002/eji.201847888

Akihiro Maeda, Elisabeth Digifico, Fernando T. Andon, Alberto Mantovani and Paola Allavena

Poly(I:C) stimulation is superior than Imiquimod to induce the antitumoral functional profile of tumor-conditioned macrophages

© 2019 The Authors. *European Journal of Immunology* published by WILEY-VCH Verlag GmbH & Co. KGaA, Weinheim.

Supl. Fig. 1.

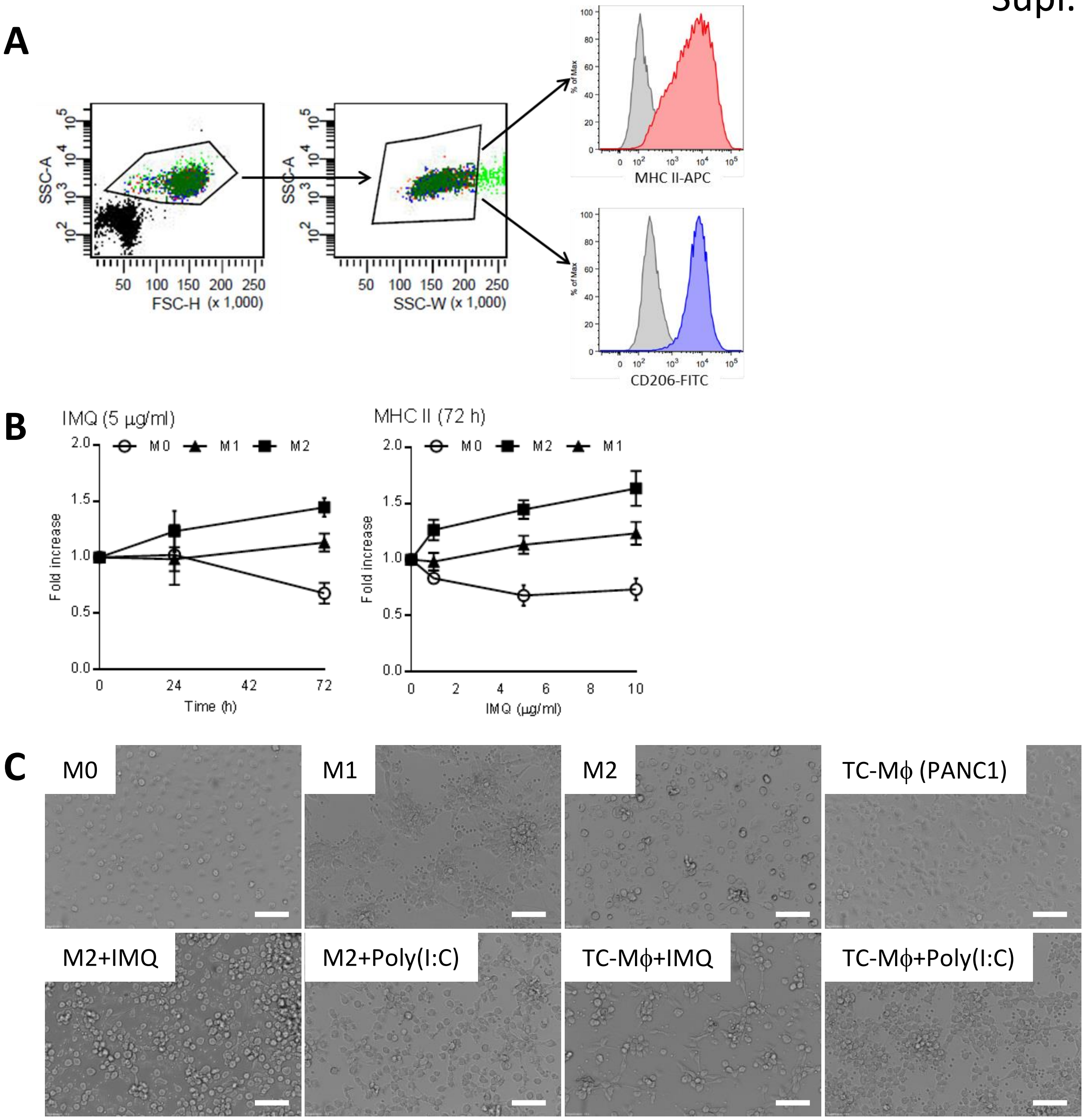

Supplementary Fig.1. Dose-response and kinetics of IMQ on differently polarized human macrophages

Macrophages were *in vitro* differentiated by stimulating monocytes with 25 ng/ml of rhM-CSF for 6 days, and then polarized with 100 ng/ml of LPS and 50 ng/ml of IFNg (M1), 20 ng/ml of IL-4 (M2) or medium (M0) for 24 hr. (A) shows the gating strategy to analyze the macrophages for flow cytometory. In histogram, non stained samples were shown in gray color. (B) Kinetics of MHC II expression (flow cytometry) in differently polarized macrophages treated with 5 mg/ml of IMQ along the indicated time points (left panel) and dose response of IMQ analyzed as MHC II up-regulation after 72 hr (right panel). Results are expressed as fold increase (Mean fluorescent intensity), relative to untreated macrophages. Two independent experiments with a total of 3 donors. (C) Morphology of differently polarized macrophages treated as indicated; IMQ: 5 mg/ml; LPS: 100 ng/ml +IFNg: 50 ng/ml. Scale bars indicate 50 mm.

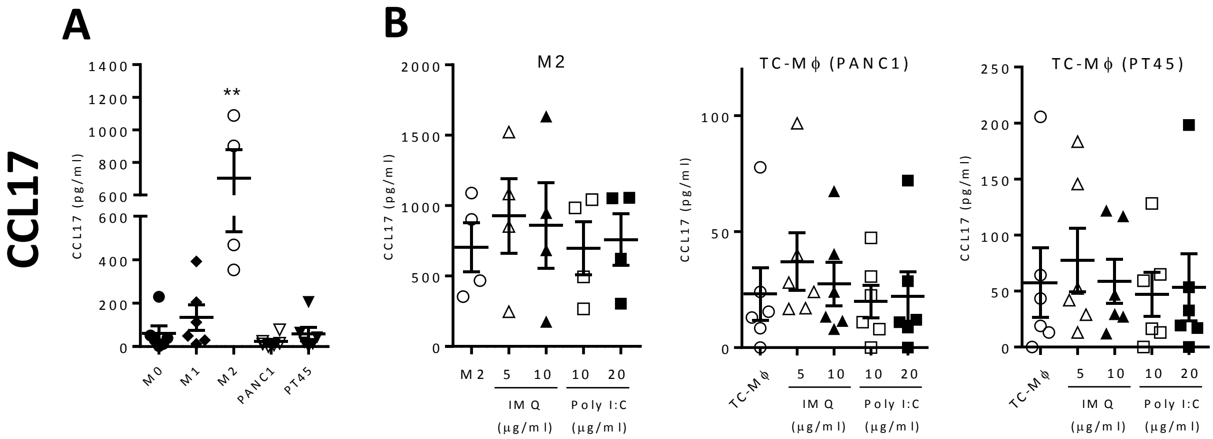

Supplementary Fig.2. CCL17 secretion by M2-macrophages and TC-Mϕ treated with IMQ or Poly(I:C).

The secretion of CCL17 was measured by ELISA from M0, M1, M2 macrophages, or TC-Mϕ treated with IMQ or Poly(I:C) for 24 hr. Results are expressed as mean ± SEM from up to three independent experiments (total 3-6 donors).

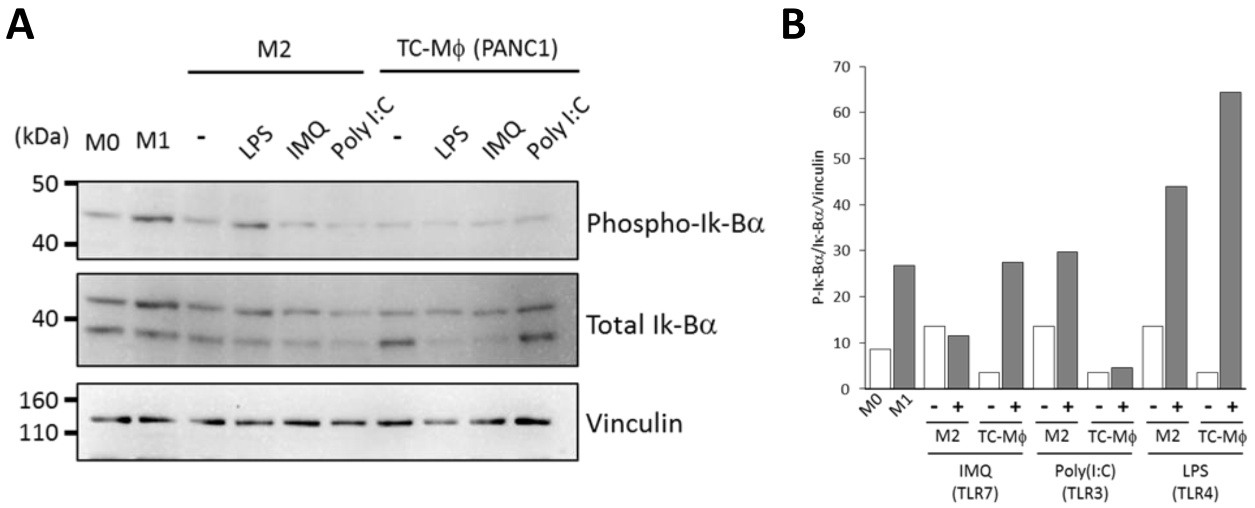

Supplementary Fig.3. Phosphorylation of Iκ-Bα upon treatment with IMQ and Poly(I:C).
(A) Western blot analysis of phosphorylated Iκ-Bα in different macrophage preparations after stimulation with IMQ (10 μg/ml) or Poly(I:C) (20 μg/ml) or LPS (100 ng/ml) for 30 min. (B) Analysis of phospho-Iκ-Bα after normalization with anti-vinculin Ab.
